# The Interplay of Genetics and Lifestyle in MASLD: Focus on LPIN1 rs13412852 and Sedentary Behaviour

**DOI:** 10.3390/ijms27041644

**Published:** 2026-02-08

**Authors:** Isabella Franco, Rossella Donghia, Antonella Bianco, Claudia Beatrice Bagnato, Nicola Verrelli, Caterina Bonfiglio, Elisabetta Di Nicola, Giovanna Forte, Martina Lepore Signorile, Marialaura Latrofa, Marika D’Addabbo, Katia De Marco, Vittoria Disciglio, Paola Sanese, Gianluigi Giannelli, Candida Fasano, Cristiano Simone, Valentina Grossi

**Affiliations:** 1 Laboratory of Movement and Wellness, National Institute of Gastroenterology—IRCCS “Saverio de Bellis”, 70013 Castellana Grotte, Italy; isabella.franco@irccsdebellis.it (I.F.); antonella.bianco@irccsdebellis.it (A.B.); claudia.bagnato@irccsdebellis.it (C.B.B.); nicola.verrelli@irccsdebellis.it (N.V.); 2 Data Science Unit, National Institute of Gastroenterology—IRCCS “Saverio de Bellis”, 70013 Castellana Grotte, Italy; rossella.donghia@irccsdebellis.it (R.D.); catia.bonfiglio@irccsdebellis.it (C.B.); 3 Medical Genetics, National Institute of Gastroenterology—IRCCS “Saverio de Bellis”, 70013 Castellana Grotte, Italy; elisabetta.dinicola@irccsdebellis.it (E.D.N.); giovanna.forte@irccsdebellis.it (G.F.); martina.lepore@irccsdebellis.it (M.L.S.); marialaura.latrofa@irccsdebellis.it (M.L.); marika.daddabbo@gmail.com (M.D.); katia.demarco@irccsdebellis.it (K.D.M.); vittoria.disciglio@irccsdebellis.it (V.D.); paola.sanese@irccsdebellis.it (P.S.); candida.fasano@irccsdebellis.it (C.F.); 4 Department of Translational and Precision Medicine, University of Rome “La Sapienza”, 00185 Roma, Italy; 5 Scientific Direction, National Institute of Gastroenterology—IRCCS “Saverio de Bellis”, 70013 Castellana Grotte, Italy; gianluigi.giannelli@irccsdebellis.it; 6 Medical Genetics, Department of Precision and Regenerative Medicine and Jonic Area (DiMePRe-J), University of Bari Aldo Moro, 70124 Bari, Italy

**Keywords:** MASLD, sedentary behaviour, single nucleotide polymorphism

## Abstract

The LPIN1 rs13412852 variant has been linked to lipid levels and liver disease in children. This genotype may modulate the liver’s response to sedentary behaviour, potentially increasing the vulnerability of certain individuals to liver dysfunction. These findings underscore the need to consider both genetic predisposition and environmental exposures when evaluating disease risk. This study aims to investigate the association between the LPIN rs13412852 T-allele and sedentary behaviour and to explore how the interplay between genetic and environmental factors may contribute to individual susceptibility to liver-related conditions. rs13412852 was genotyped in a cohort from Southern Italy (n = 394), and all participants were administered an International Physical Activity Questionnaire (IPAQ), collected a blood sample, and underwent an abdominal ultrasound analysis. The association between metabolic dysfunction-associated steatotic liver disease (MASLD), rs13412852, and sedentary behaviour, alone and together with interaction, was studied. The results indicated a statistical association on MASLD, of rs13412852, and sedentary levels (OR = 1.80, 1.06 to 3.05 95% C.I., *p* = 0.03, and OR = 1.72, 1.13 to 2.64 95% C.I.), respectively, and also with interaction between moderate or sever sedentary level and T-carrier (OR = 2.99, 1.39 to 6.45 95% C.I., *p* = 0.005) adjusted for some covariates. The risk of MASLD was highest among individuals with both moderate/severe sedentary behaviour and the CT/TT genotype, suggesting a potential synergistic effect. These findings establish LPIN1 as both a physiological gatekeeper and a genetic susceptibility locus, with its influence subject to modification via behavioural treatments.

## 1. Introduction

Metabolic dysfunction-associated steatotic liver disease (MASLD) is one of the most common chronic liver diseases, affecting approximately 30% of the adult population worldwide, with its prevalence set to increase [1]. The high socioeconomic impact of MASLD represents a serious public health issue that must be addressed by medical societies and policymakers [2]. The term MASLD was proposed and adopted in 2023 in a Delphi consensus statement [3] drafted by several scientific societies, effectively replacing the previous terms, non-alcoholic fatty liver disease (NAFLD) and metabolic-associated fatty liver disease (MAFLD), and is integrated into the new consensus definition of hepatic steatosis (SLD) [4]. MASLD is characterized by excessive fat accumulation in the liver [5] and is associated with cardiovascular risk factors, including obesity, sedentary lifestyle, low physical activity, and genetic and environmental factors [6]. The new definition includes several conditions, including isolated hepatic steatosis (metabolic-associated liver disease, MASL), metabolic-associated steatohepatitis (MASH), fibrosis, cirrhosis, and the development of hepatocellular carcinoma (HCC) [7,8]. The criteria for diagnosing MASLD have also changed. At least one of five cardiometabolic risk factors must be present in an individual with documented steatosis and no other recognizable cause [3]. Even moderate alcohol consumption (an average daily intake of 20–50 g for women and 30–60 g for men) is an important factor to consider in MASLD, but it is no longer an exclusion criterion, as in the previous definition of NAFLD [3,9]. The development of MASLD is strongly influenced by the combined effects of low levels of physical activity and unhealthy diets; therefore, it is essential to intervene in lifestyle [10,11,12]. Physical activity plays a key role in managing MASLD because, when performed regularly, it reduces liver fat and improves overall metabolic health [13]. Numerous randomized clinical trials (RCTs) and meta-analyses have shown that exercise alone, without dietary interventions or significant weight loss, reduces hepatic steatosis in individuals with MASLD [14]. Various training programmes, including aerobic training, resistance training, high-intensity interval training (HIT), or a combination of these, with different session frequencies, durations, and intensities, have been tested for the management of hepatic steatosis. Most of these are effective in reducing steatosis [15,16]. Among the different types of training, moderate-intensity aerobic exercise is the most effective. In fact, it is known that practicing it for at least 150 min per week can reduce liver fat accumulation, slowing the progression of the disease, improving insulin sensitivity, promoting weight loss, and exerting positive effects on the cardiovascular system [17,18,19]. Aerobic training, whether high-intensity interval training (HIIT) or continuous moderate-intensity training, is equally effective in reducing liver fat [20]. Physical exercise not only reduces hepatic steatosis and visceral fat and improves muscle mass and function even in the advanced stages of liver disease but also improves quality of life and cardiorespiratory capacity [21,22].

Leisure-time physical activity (LTPA) is also crucial for managing and reducing MASLD, as individuals who engage in more recreational physical activity have higher long-term survival rates than sedentary individuals [23]. It is interesting to note, however, that sedentary behaviour has been identified as a modifiable risk factor for mortality independent of time spent in physical activity [24]. Several studies have suggested that sedentary behaviour is associated with obesity, diabetes, insulin resistance, metabolic syndrome, cardiovascular disease, and cancer [25,26]. Furthermore, it has been shown that the amount of LTPA and the amount of time spent sitting (TSS) may play a role in the development and progression of NAFLD [27] and are associated with an increased risk of MASLD progression [28].

While lifestyle-related factors such as physical inactivity have been extensively studied and recognized as key contributors to the development of MASLD, increasing evidence suggests that genetic predispositions also play a crucial role in both the onset and progression of the disease, highlighting the multifactorial nature of its pathogenesis.

Indeed, the role of genetic variability in MASLD, particularly single-nucleotide polymorphisms (SNPs), has been the focus of extensive research in recent years. SNPs of several genes have been associated with liver damage because they are involved in lipid metabolism (e.g., Patatin-like phospholipase domain containing-3 [PNPLA3] and Phosphatidate phosphatase LPIN1[LPIN1]) [29], oxidative stress (e.g., Superoxide dismutase 2 [SOD2]) [30], and fibrogenesis (e.g., Kruppel-like factor 6 [KLF6]) [31].

LPIN1 acts as a cytosolic and ER-associated phosphatidate phosphatase (PAP1), facilitating the Mg^2^-dependent hydrolysis of phosphatidic acid (PA) to yield diacylglycerol (DAG). This reaction represents a rate-limiting step in the glycerolipid biosynthetic pathway, acting as a biochemical junction between the synthesis of triglycerides (TAGs) and key phospholipids such as phosphatidylcholine (PC) and phosphatidylethanolamine (PE) [32,33].

LPIN1 is a large, evolutionarily conserved protein characterized by two functionally separated domains: the N-terminal Lipin (N-LIP) domain and the C-terminal Lipin (C-LIP) domain. The C-LIP domain comprises the catalytic DXDX(T/V) motif, a characteristic sequence for Mg^2^-dependent phosphatidate phosphatase (PAP) activity, which hydrolyzes phosphatidic acid to diacylglycerol [32,33]. The N-LIP domain facilitates protein–protein interactions, membrane connection, and nuclear translocation. This area encompasses a polybasic motif that promotes binding to anionic lipids, such as phosphatidic acid (PA), and may function as a nuclear localization signal (NLS) [34].

LPIN1, a phosphatase highly expressed in adipose tissue and the liver, is involved in metabolic pathways linking adipose tissue to the liver [35].

LPIN1 SNPs and haplotypes have been associated with several components of metabolic syndrome [36]. Moreover, there is growing awareness that the expression of the LPIN1 variant rs13412852 may be age-dependent, i.e., that the phenotype may be more or less marked or involve different traits during the developmental age. Indeed, the LPIN1 variant rs13412852 was associated with lipid levels, NASH severity, and hepatic fibrosis in children with NAFLD. In contrast, it influenced body mass, but not the severity of liver histology, in adults with NAFLD of the same ethnicity [37,38].

The genotype may influence the liver’s response to sedentary behaviour, making some people more vulnerable to liver problems. This highlights the importance of considering both genetic and environmental factors when assessing disease risk.

The aim of our study is to evaluate the association between the LPIN rs13412852 T-allele and sedentary behaviour, considering how the outcomes of genetic and environmental factors may influence susceptibility to liver health problems.

## 2. Results

The demographic, clinical, genetic, and biochemical characteristics of the total cohort stratified for sedentary level are reported in Table 1, considering the baseline characteristics of the covariates included in the association models.

According to the IPAQ, 78.68% of participants had a low sedentary profile, with a mean age of 53.93 ± 8.81 years and a male prevalence of 37.82%. Participants in the moderate/high sedentary category were significantly younger than those in the low sedentary group (51.90 ± 9.35 vs. 54.48 ± 8.59 years, *p* = 0.01), and a significant association was observed with educational level (*p* < 0.001). Among blood parameters, only HDL cholesterol showed a statistically significant difference, with lower levels in the more sedentary group (48.68 ± 11.51 vs. 52.19 ± 12.69 mg/dL, *p* = 0.02).

**Figure 1:**
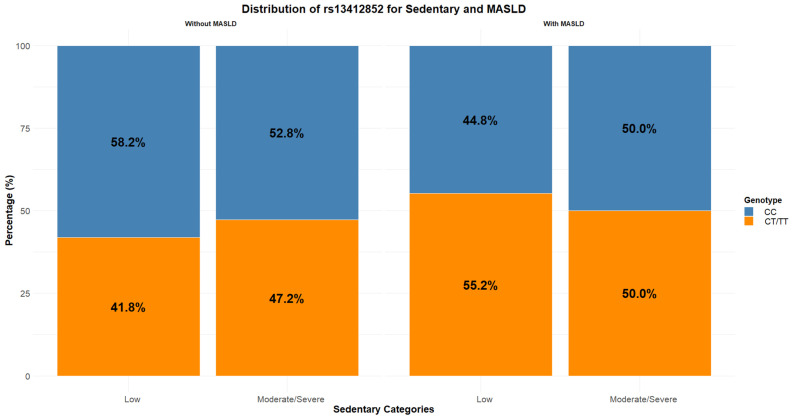
Distribution of rs13412852 into the sedentary and MASLD groups.

When examining the prevalence of sedentary behaviour and genotype within the MASLD variable, no statistically significant differences were observed; however, a trend emerged in the group without MASLD (Figure 1).

**Figure 2:**
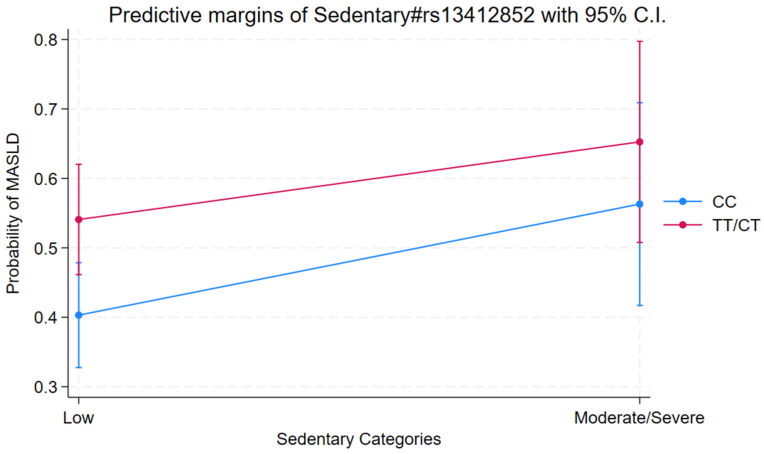
Predictive margins from the model of MASLD using sedentary categories and genotypes.

As sedentary behaviour increased (from ‘Low’ to ‘Moderate/Severe’), the proportion of individuals with the ‘CC’ genotype slightly decreased (from 58.2% to 52.8%), while the prevalence of the ‘CT/TT’ genotype increased (from 41.8% to 47.2%). This trend was not observed in individuals with MASLD. These findings suggest a potential association between the CT/TT genotype and higher levels of sedentary behaviour, even in the absence of liver disease (Figure 2). Logistic regression analysis revealed a significant association between sedentary behaviour, genotype rs13412852, and the risk of MASLD. Individuals with moderate/severe sedentary behaviour showed a higher risk of MASLD compared to those with low sedentary levels (reference category) (OR = 1.80; *p* = 0.03, 95% C.I. 1.06 to 3.05). Additionally, the CT/TT genotype was independently associated with increased odds of MASLD compared to the CC genotype (OR = 1.72; *p* = 0.01, 95% C.I. 1.13 to 2.64). Importantly, the interaction term between sedentary behaviour and genotype was also significant. The risk of MASLD was highest among individuals with both moderate/severe sedentary behaviour and the CT/TT genotype (OR = 2.99, *p* = 0.005, 95% C.I. 1.39 to 6.45), suggesting a potential synergistic effect (Table 2).

Figure 2 shows the predicted margins for MASLD probability across sedentary categories and genotypes. A steeper increase in MASLD probability is observed in individuals with the CT/TT genotype as sedentary behaviour increases, while the increase is more modest in those with the CC genotype. These findings support a possible gene–environment interaction, whereby individuals carrying the CT/TT genotype may be more susceptible to the effects of sedentary behaviour on liver health.

## 3. Discussion

MASLD represents a significant global health challenge due to its increasing prevalence, which imposes a substantial burden on healthcare systems, negatively impacts patient well-being, and results in considerable economic costs [39]. Addressing MASLD requires a thorough comprehension of its interrelated factors, encompassing its prevalence, healthcare burden, and economic implications. Recent data indicate that MASLD is the most common chronic liver disease globally, emphasizing the close relationship between systemic metabolic dysregulation and hepatic lipid accumulation [39]. In particular, the excessive hepatocyte storage of triglycerides (TAGs) resulting from enhanced de novo lipogenesis, impaired fatty acid β-oxidation, and inefficient lipid export is a hallmark of MASLD [40,41]. Although these mechanisms have been thoroughly described, there has been comparatively less focus on the intracellular factors that influence the distribution of lipid intermediates. In this context, LPIN1 may be considered as a biochemical switch that could influence hepatic lipid fate and the progression of disease.

Functionally, LPIN1 acts as a magnesium-dependent phosphatidate phosphatase type 1 (PAP1) that facilitates the dephosphorylation of phosphatidic acid (PA) into diacylglycerol (DAG), a key intermediate in both TAG biosynthesis (through diacylglycerol O-acyltransferase 1 and 2 [DGAT1/2]) and phospholipid synthesis via the Kennedy pathway, resulting in the production of phosphatidylcholine and phosphatidylethanolamine [33,42]. The dual metabolic branching point designates LPIN1 as a regulatory element that equilibrates energy storage as neutral lipids with the production of membrane lipids essential for organelle integrity and vesicular trafficking. In hepatocytes, excessive diversion of DAG toward TAG synthesis may lead to lipid droplet accumulation and cellular lipotoxicity, thereby contributing to the progression of MASLD [43].

Under conditions of metabolic stress (fasting), LPIN-1 is a key transcriptional coactivator of genes involved in fatty acid degradation by interacting with Peroxisome proliferator-activated receptor alpha (PPARα) and Peroxisome proliferator-activated receptor gamma coactivator 1-alpha (PGC-1α) [44].

LPIN1 is predominantly expressed in adipose tissue, skeletal muscle, and liver, where it governs tissue-specific lipid flux and constitutes the predominant source of PAP activity. It is also present in the heart, kidney, and brain, though at reduced concentrations [35,44].

In adipose tissue, LPIN1 plays a crucial role in TAG synthesis and lipid storage. The enzyme facilitates the transformation of PA into diacylglycerol DAG, which is a crucial process in the glycerol-3-phosphate pathway, enabling adipocytes to accumulate fatty acids as triacylglycerols [35]. Additionally, LPIN1 promotes adipocyte differentiation through its transcriptional coactivator activity, thereby enhancing the expression of peroxisome proliferator-activated receptor gamma (PPARγ), a key regulator of adipogenesis [35]. Interestingly, in adipocyte-specific LPIN1 knockout mice (Adn Lpin1^−^/^−^), the absence of LPIN1 markedly decreased adipose tissue mass. These mice exhibited compensatory overexpression of LPIN2 while maintaining negligible PAP activity, underscoring the predominant function of LPIN1 in adipose lipid storage and systemic energy balance [45].

In skeletal muscle, LPIN1 facilitates mitochondrial fatty acid oxidation and maintains the cellular energetic homeostasis. The genetic ablation of LPIN1 specifically in the muscle of mice results in mitochondrial malfunction, altered respiratory capacity, and impaired lipid consumption [46]. These traits align with LPIN1’s role as a transcriptional co-activator of PGC-1α and PPARα, which govern oxidative metabolism. Furthermore, fat-laden (fld) animals, which are deficient in functional LPIN1, exhibit muscle glycogen buildup and energy dysregulation, underscoring the significance of LPIN1 in muscle fuel selection and lipid metabolism [47].

LPIN1 modulates triglyceride production and the secretion of very-low-density lipoprotein (VLDL) in the liver. Hepatic LPIN1 facilitates TAG synthesis via its PAP activity and concurrently influences lipid-related gene expression in the nucleus. LPIN1-KO murine hepatocytes result in a substantial reduction in adipose fat mass [48]. Furthermore, LPIN1 regulates VLDL-TAG export via transcriptional pathways rather than solely through its enzymatic activity. In LPIN1-deficient fld mice, diminished VLDL production is correlated with enhanced insulin sensitivity, suggesting a role in hepatic lipid export and insulin signalling [44].

Although no direct correlations with physical activity levels have yet been found for most lipids, elevated blood TAG levels appear to be associated with a prolonged sedentary lifestyle [48,49,50]. In fact, physical activity increases the lipolytic rate while maintaining a low concentration of triglycerides in the blood [51]. Several lines of evidence suggest that SNPs may exert functional effects by modulating transcriptional regulation, splicing efficiency, mRNA stability, or protein abundance [52]. Consistent with the literature, we hypothesize that rs13412852 may influence LPIN1 expression levels, potentially by creating or modifying regulatory elements (e.g., enhancer activity), thereby increasing LPIN1 protein levels. Functionally, this effect would be particularly relevant in the context of sedentary behaviour. Recent evidence demonstrates that short-term physical inactivity (e.g., 24 h limb immobilization) directly increases LPIN1 expression in humans and promotes its phosphatase (PAP) activity in animal models, thereby impairing insulin-stimulated 2-deoxyglucose uptake [53]. This activation leads to intramuscular accumulation of diacylglycerol (DAG) and subsequent insulin resistance via PKCε activation [53]. Given that rs13412852 has been associated with elevated insulin levels and responsiveness to insulin sensitizers, we hypothesize that this polymorphism may enhance LPIN1 transcription under sedentary conditions [54]. Finally, LPIN1 protein stability is tightly regulated by metabolic signalling pathways; in particular, AKT1 promotes LPIN1 stabilization, while LKB1 promotes its proteasomal degradation. Since AKT1 activity is enhanced by insulin signalling, and the rs13412852 variant has been associated with increased insulin levels and increased insulin sensitivity, the polymorphism could promote LPIN1 transcription under conditions of physical inactivity [54]. These mechanisms are hypothetical and require further experimental validation, yet they provide a coherent biological framework to explain how rs13412852 may modulate the phenotypic response to sedentary behaviour.

Collectively, these results highlight LPIN1’s role as a metabolic integrator, orchestrating lipid synthesis, storage, and oxidation in a tissue-specific context. Dysfunction of this LPIN-1-dependent system contributes to systemic metabolic illnesses, including obesity, insulin resistance, and MASLD, positioning it as a possible therapeutic target for metabolic disease intervention.

Our results showed a significant association between moderate to severe sedentary behaviour (SB), the CC/CT genotype, and the risk of MASLD. It is now well known that SB is an independent risk factor for numerous chronic diseases, including obesity, type 2 diabetes, cardiovascular disease, and certain cancers, even in physically active individuals [55,56,57,58,59]. Scientific evidence suggests that a highly sedentary lifestyle compromises the metabolic benefits of physical exercise, indicating that time spent sitting not only directly affects health but also modulates the effectiveness of physical activity [60,61,62] and reduces its protective effects [63]. It is therefore essential to intervene not only by increasing physical activity but above all by reducing and interrupting sedentary time, promoting the concepts that “every movement counts” and “a little is better than nothing,” as endorsed by the World Health Organization (WHO) [64]. In this context, our results add an important piece to the puzzle. Analysis of the genotype-environment interaction revealed an almost threefold risk of MASLD in sedentary individuals with a risk genotype, suggesting a synergistic effect. This indicates that genetic predisposition does not act in isolation but can be amplified by inadequate lifestyles. From a clinical and preventive perspective, these findings reinforce the importance of targeting sedentary behaviour, particularly in individuals who may be genetically susceptible, thereby highlighting physical inactivity as a key modifiable risk factor.

The metabolic effects observed are consistent with pathways involving LPIN1, given its role in dephosphorylating phosphatidic acid (PA) to diacylglycerol (DAG), a critical step in triglyceride biosynthesis. However, while the rs13412852 variant has been associated with metabolic phenotypes, its direct functional impact on LPIN1 expression or enzymatic activity has not yet been established. Therefore, any proposed link between this polymorphism and increased lipid accumulation should be regarded as speculative and remains to be confirmed by dedicated functional studies.

Mechanistically, it is plausible that LPIN1 rs13412852 disrupts DAG partitioning, decreases fatty acid oxidation, and increases lipogenesis in a sedentary lifestyle, thereby promoting the development of steatosis. These findings establish LPIN1 as both a physiological gatekeeper and a genetic susceptibility locus, with its influence subject to modification via behavioural treatments.

## 4. Materials and Methods

### 4.1. Study Population

**Figure 3:**
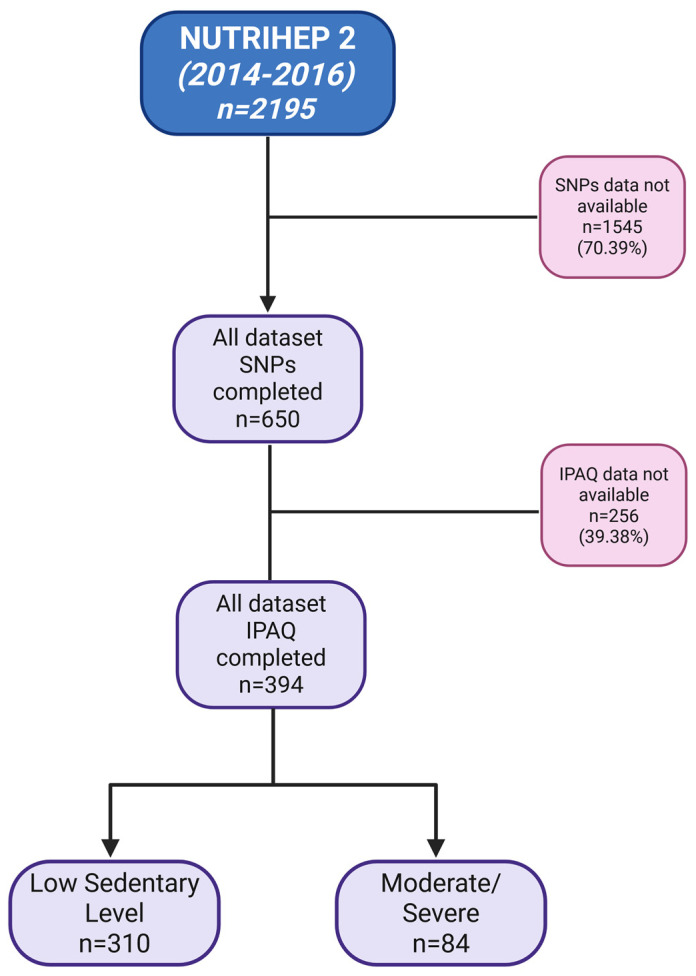
Flowchart of the cohort study. Image created in BioRender. Rossella Donghia. (2025) http://BioRender.com/68711df81287dd9568cd0195 (accessed on 11 July 2025).

The NUTRIHEP study is a cohort derived from the medical records of general practitioners in the municipality of Putignano (Bari), a small town in Southern Italy located approximately 20 km from the coast. Between 30 June 2004 and 30 June 2005, trained physicians conducted interviews with 2550 participants (aged > 18 years) to collect data on sociodemographic characteristics, clinical history, and dietary habits [65]. The participants followed the same protocol at baseline enrollment. From 30 June 2014 to 30 June 2018, all eligible individuals, starting with those previously enrolled in the first follow-up, were invited to take part in the follow-up phase. The participation rate was 86.08% (n = 2195). Among these, 650 biological samples were stored for future molecular analyses and included in the present study, and of these, 394 (60.61%) were administered the International Physical Activity Questionnaire (IPAQ) (Figure 3).

All participants signed written informed consent after receiving information on the medical data recorded. Ethical committee approval was not required for the baseline enrollment phase nor for the first recall. However, approval from the Ethics Committee was obtained for the second recall, and such approval was granted with retroactive validity for all preceding phases. This study was approved by the Ethics Committee of the Ministry of Health (updated Ethics Committee, Prot. 368 of 16 April 2025). Overall mortality was updated on 31 December 2024.

### 4.2. Lifestyle, Clinical, and Dietary Assessment

Lifestyle and anthropometric evaluations were conducted by clinicians during visits to the study centre. Smoking status was determined based on the single question, “Do you smoke?” Educational level was reported in terms of years of schooling. Weight was measured using an electronic scale (SECA^©^) and recorded to the nearest 0.1 kg, while height was assessed with a wall-mounted stadiometer (SECA^©^) and recorded to the nearest 1 cm. Body mass index (BMI) was calculated as weight in kilograms divided by height in metres squared (kg/m^2^). Venous blood samples were drawn from all participants in the morning following an overnight fast. Samples were processed by trained personnel and stored as aliquots in a biobank, following validated protocols. Categorical variables for blood parameters (e.g., out of range) were defined according to the normal reference ranges provided by the laboratory performing the assays.

Steatosis has been diagnosed in subjects with hepatic steatosis on ultrasound [66], and diabetes was diagnosed based on ongoing pharmacological treatment or confirmation by an endocrinologist. MASLD was defined according to the diagnostic criteria established by the Delphi consensus [3]. The International Physical Activity Questionnaire—Long Form (IPAQ-LF) [67] was self-administered in all studies, with staff available to assist with reading the questionnaire if needed. Sedentary behaviour was assessed by asking participants to report the average number of hours per day spent sitting or lying down, excluding sleep. The reported daily value was multiplied by seven to obtain the total number of sedentary hours per week. Based on this measure, participants were categorized into two groups: low sedentary behaviour (<35 h/week, corresponding to <5 h/day) and moderate/severe sedentary behaviour (≥35 h/week).

### 4.3. DNA Extraction and TaqMan PCR Assay

Genomic DNA was extracted from peripheral blood with the MagCore^®^ Genomic DNA Whole Blood Kit (MGB400-08, Amerigo Scientific, Central Islip, NY, USA) according to the manufacturer’s instructions. Genotyping LPIN rs13412852 was performed using TaqMan SNP Genotyping Assays (ID: C__32194351_10 Applied Biosystems, Waltham, MA, USA). Briefly, 5 ng of DNA was amplified for each sample in a total volume of 10 μL using TaqMan™ Genotyping Master Mix (4371353, Applied Biosystems) according to the manufacturer’s instructions. The primer and probe sequences, as well as the PCR conditions, are available upon request (https://www.thermofisher.com/pl/en/home.html, accessed on 1 June 2024). Randomly selected samples were verified using Sanger sequencing.

### 4.4. Statistical Analysis

The subjects’ characteristics are reported as mean and standard deviation (M ± SD) for continuous variables and as frequency and percentages (%) for categorical variables. The Wilcoxon rank sum test was used to evaluate the differences in parameters between sedentary levels for continuous variables, while the chi-square or Fisher’s (when necessary) test was used for categorical variables. We estimated a multiple logistic regression model with MASLD as the dependent variable (yes vs. no) and sedentary categories as predictors. The models were also adjusted for some covariates (age and gender), and estimated coefficients were transformed into odds ratios (OR) and relative confidence intervals at 95%. Stratified models were built for gender. Margins were calculated from predictions of a previously fitted model at fixed values of covariates, and after, a marginsplot was built.

To test the null hypothesis of non-association, the two-tailed probability level was set at 0.05. The analyses were conducted with StataCorp. 2025. Stata Statistical Software: Release 19. College Station, TX: StataCorp LLC, and RStudio “Mariposa Orchid” Release (ab7c1bc7, 1 June 2025). The statistical analysis was reviewed to be consistent with the Checklist for Statistical Assessment of Medical Papers statement [68].

## 5. Conclusions

These results showed that LPIN1 bridges the metabolic decision between phospholipid and triglyceride synthesis and that its dysregulation—through altered expression, enzymatic activity, or genetic variation—may significantly contribute to the pathogenesis of MASLD. The LPIN1 rs13412852 variant, particularly in the context of low physical activity, appears to increase this risk. Despite these results, our study’s findings should be interpreted with caution due to certain constraints. One limitation is the potential underestimation or overestimation of physical activity data collected through the IPAQ. This bias may stem from a tendency to provide socially desirable responses or from an inaccurate self-assessment of individual activity levels, which could influence the strength of the observed associations. Although objective tools, such as accelerometer-based monitors, are becoming increasingly important for standardizing physical activity assessments [69], the IPAQ continues to be a widely used and practical instrument in large population studies. Another limitation is that the analysis focused on a single functional SNP (rs13412852), as well as sedentary behaviour and its relationship to MASLD risk.

Overall, it seems reasonable to assume that no single gene variant may be sufficient to accurately stratify risk, while predictive power would be improved by analyzing several SNPs or combining their effects into a genetic risk score. Of note, it would be important to demonstrate the biological role of this SNP and how it, in association with sedentary behaviour, contributes to susceptibility to liver-related conditions. To this end, it would be useful to perform experiments on mouse models in the laboratory. Finally, the observational nature of this study prevents the establishment of causal inferences or the formulation of clinical recommendations regarding physical activity. The aim was primarily to explore the association between sedentary behaviour and MASLD. Further longitudinal and experimental studies are required to clarify the biological role of this SNP and to investigate whether increased physical activity may influence the susceptibility of certain individuals to liver dysfunction. Based on this evidence, future studies investigating LPIN1’s regulatory network in human liver tissue, its interaction with lifestyle factors, and its potential as a therapeutic target or biomarker are warranted.

## Figures and Tables

**Table 1:** Demographic and clinical characteristic parameters in the NUTRIHEP cohort between sedentary levels (n = 394).

Parameters *	Sedentary		*p* ^^^
	Low (n = 310)	Moderate/Severe (n = 84)	
Age (yrs)	54.48 ± 8.59	51.90 ± 9.35	0.01
Gender (M)	112 (36.13)	37 (44.05)	0.18 ^†^
Education (%)			<0.001 ^†^
None	1 (0.34)	0 (0.00)	
Elementary School	57 (19.39)	5 (6.17)	
Secondary School	122 (41.50)	28 (34.57)	
High School	96 (32.65)	30 (37.04)	
Degree	9 (3.06)	7 (8.64)	
Post-Degree	9 (3.06)	11 (13.58)	
Civil Status (%)			0.21 ^†^
Single	26 (8.84)	13 (16.05)	
Married or Cohabiting	244 (82.99)	65 (80.25)	
Divorced or Separated	11 (3.74)	1 (1.23)	
Widower	13 (4.42)	2 (2.47)	
Smoker (Yes) (%)	42 (14.24)	7 (8.64)	0.18 ^†^
Waist Circumference (cm)	90.92 ± 12.41	91.93 ± 11.73	0.33
Hip Circumference (cm)	102.64 ± 9.16	102.98 ± 8.34	0.57
BMI (kg/m^2^)	27.66 ± 4.78	27.64 ± 4.06	0.78
Kcal	2152.78 ± 748.55	2202.83 ± 693.42	0.52
SNP rs13412852 (%)			0.90 ^†^
CC	161 (51.94)	43 (51.19)	
CT/TT	149 (48.06)	41 (48.81)	
Clinical			
Systolic Blood Pressure (mmHg)	121.18 ± 14.88	118.09 ± 13.12	0.14
Diastolic Blood Pressure (mmHg)	78.93 ± 7.94	78.51 ± 7.52	0.50
Hypertension (Yes) (%)	89 (30.17)	24 (29.63)	0.92 ^†^
Diabetes (Yes) (%)	13 (4.19)	3 (3.57)	0.80 ^†^
MASLD (Yes) (%)	145 (46.77)	48 (57.14)	0.09 ^†^
Blood			
Glycemia (mg/dL)	96.30 ± 14.96	96.69 ± 12.98	0.80
HOMA	1.73 ± 1.14	2.04 ± 1.42	0.07
Insulin (mmol/L)	7.10 ± 3.99	8.35 ± 5.03	0.04
Cholesterol (mg/dL)	198.70 ± 35.13	195.58 ± 35.84	0.55
Triglycerides (mg/dL)	98.64 ± 61.98	103.69 ± 60.13	0.34
HDL (mg %)	52.19 ± 12.69	48.68 ± 11.51	0.02
RBC (10^6^/µL)	5.01 ± 0.51	4.98 ± 0.59	0.56
Hemoglobin (g/dL)	14.07 ± 1.42	14.02 ± 1.53	0.76
Hematocrit (L/L)	42.26 ± 3.65	42.20 ± 3.83	0.81
MCV (fL)	84.77 ± 7.50	85.25 ± 6.55	0.76
MCH (pg)	28.23 ± 2.84	28.32 ± 2.52	0.86
MCHC (g/dL)	33.26 ± 1.03	33.19 ± 1.12	0.65
RDW-CV (%)	13.81 ± 1.58	13.65 ± 1.14	0.71
Platelets (10^3^/µL)	232.99 ± 52.27	233.73 ± 44.79	0.90
WBC (10^3^/µL)	5.68 ± 1.60	5.82 ± 1.79	0.35
Neutrophils (%)	57.10 ± 7.56	58.15 ± 8.03	0.40
Lymphocytes (%)	32.32 ± 6.87	31.29 ± 8.25	0.36
Monocytes (%)	7.24 ± 1.67	74.22 ± 1.47	0.74
Basophils (%)	0.53 ± 0.27	0.51 ± 0.24	0.62
Neutrophils (10^9^/L)	3.28 ± 1.17	3.42 ± 1.33	0.30
Lymphocytes (10^3^/µL)	1.80 ± 0.53	1.78 ± 0.69	0.48
Monocytes (10^3^/µL)	0.41 ± 0.14	0.42 ± 0.15	0.49
Basophils (10^3^/µL)	0.03 ± 0.01	0.03 ± 0.01	0.84
Eosinophils (10^3^/µL)	0.16 ± 0.13	0.16 ± 0.12	0.89
HbA1c (mmol/mol)	36.52 ± 6.57	36.36 ± 6.59	0.77
Total Bilirubin (mg/dL)	0.70 ± 0.34	0.68 ± 0.35	0.28
Direct Bilirubin (mg/dL)	0.17 ± 0.05	0.16 ± 0.05	0.84
GOT (U/L)	21.71 ± 5.40	21.53 ± 5.90	0.66
SGPT (U/L)	22.77 ± 11.12	23.23 ± 11.96	0.68
GGT (U/L)	16.88 ± 10.06	16.83 ± 9.69	0.85
Alkaline Phosphatase (U/L)	55.47 ± 14.93	53.76 ± 14.96	0.46
Albumin (g/dL)	4.11 ± 0.28	4.14 ± 0.28	0.32
Iron (µg/dL)	94.26 ± 30.83	90.50 ± 34.51	0.22
Ferritin (ng/mL)	94.43 ± 83.71	96.02 ± 85.37	0.73
Ceruloplasmin (mg/dL)	31.24 ± 6.79	29.61 ± 6.06	0.07
α1AT (mg/dL)	170.33 ± 26.87	166.61 ± 29.14	0.18

* As mean and standard deviation (M ± SD) for continuous variables, and as frequency and percentage (%) for categorical variables. ^ Wilcoxon rank-sum test or † chi-Square or Fisher test where necessary. Abbreviations: BMI, body mass index; MASLD, metabolic dysfunction-associated steatotic liver disease; HOMA, homeostatic model assessment; HDL, high-density lipoprotein; RBC, red blood cell; MCV, mean corpuscular volume; MCH, mean corpuscular hemoglobin; MCHC, mean corpuscular hemoglobin concentration; RDW-CV, red cell distribution width-coefficient of variation; WBC, white blood cell; GOT, aspartate amino transferase; SGPT, serum glutamic pyruvic transaminase; HbA1c, hemoglobin A1c; GGT, gamma-glutamyl transferase; and α1AT, alpha−1-antitrypsi.

**Table 2:** Logistic regression models ^ on MASLD on sedentary, SNP, and the interaction between both.

Models	OR	se (OR)	*p*	95% C.I.
Sedentary Levels				
Low [Ref.]	--	--		--
Moderate/Severe	1.80	0.48	0.03	1.06 to 3.05
rs13412852				
CC [Ref.]	--	--		--
CT/TT	1.72	0.37	0.01	1.13 to 2.64
Sedentary#rs13412852				
Low# rs13412852 (CC) [Ref.]	--	--		--
Low# rs13412852 (CT/TT)	1.81	0.44	0.01	1.12 to 2.93
Moderate/Severe# rs13412852 (CC)	2.00	0.73	0.06	0.97 to 4.10
Moderate/Severe# rs13412852 (CT/TT)	2.99	1.07	0.005	1.39 to 6.45

^ Adjusted for age, gender, and kcal. Abbreviations: OR, odds ratio; se (OR), standard error of (OR); 95% C.I., confidence interval at 95%.

## Data Availability

The original data presented in this study are openly available in FigShare at https://doi.org/10.6084/m9.figshare.29931269.
